# Nurses’ perceptions of involving family members in the care of mental health care users

**DOI:** 10.4102/curationis.v47i1.2538

**Published:** 2024-07-05

**Authors:** Nkhensani F. Mabunda

**Affiliations:** 1 Department of Nursing, Faculty of Health Care Sciences, Sefako Makgatho Health Science University, Pretoria, South Africa

**Keywords:** family involvement, long-term mental health care, management of psychiatric patients, mental illness recovery, psychiatric practice

## Abstract

**Background:**

Family involvement in mental health care is a therapeutic intervention in the management of mental illness. The global concern in long-term mental health is that families find it difficult to accept mental illness when their loved ones are admitted to receive care, treatment and rehabilitation.

**Objectives:**

To describe nurses’ perceptions of involving family members in the care of mental health care users in long-term institutions.

**Method:**

A quantitative descriptive design was used. The population comprised nurses working at three mental health institutions (MHIs). Probability simple random sampling was used to select 360 respondents. Data were collected using self-administered questionnaires.

**Results:**

The findings revealed that most (86.9%) of the nurses acknowledged that challenges affect families’ involvement in mental health care. A total of 91.4% of nurses complained that family members’ involvement was insufficient and (80.6%) indicated that poor family contact affects the provision of quality mental health care. Therefore, the respondents believed that the families’ involvement has an impact on the management of mental illness.

**Conclusion:**

Engaging family members in mental health care helps both health professionals and families to participate in patient-centred care and mental health care services. However, MHCUs benefit when their families are involved.

**Contribution:**

The study contributed to mental health nursing as its results can be used to measure the quality of health services improvements, by involving the family members during hospitalisation of their loved ones for mental health care.

## Introduction

Family involvement is an important interdisciplinary initiative that promotes the recovery from mental illness (De Corte et al. 2023:313). The World Health Organization (WHO) emphasised family to be involved in mental health in the Comprehensive Mental Health Action Plan 2018–2030 to provide information to improve the overall mental health and well-being of the family member who is admitted to a hospital to receive health care services (World Health Organization 2019:10). World Health Organization Mental Health Atlas (2020:88) and World Health Organization (2022:22) revealed that when one family member is having a mental health condition, their families and society are affected. Therefore, we adopted guidelines to integrate mental health into primary health care that include ongoing psychotherapy interventions for mental health disorders.

In long-term mental health care, treatment and rehabilitation, intervention such as psychotherapy is known to be a specialised treatment proposed to treat mental illness for over 100 years (Pawlak & Kacprzyk-Straszak 2020:25). Psychotherapy is an intervention that is planned for both patients and their families to reduce the length of admission and have a positive effect on mental health nurses who often undergo high levels of burnout while rendering mental health care services (Berry et al. 2022:11).

According to Tambling, Russell and D’Aniello (2021:1665), psychotherapy is initiated to engage family members regularly to understand the treatment plan, actively participate and support their loved ones diagnosed with mental illness to receive care, treatment and rehabilitation in long-term mental health institutions (MHIs). In this regard, family members recognise the patient’s progress and prepare them to be ready for the discharge of their loved one. The authors emphasised that discharge planning addresses the practical reasons for hospitalisation to receive care, treatment and rehabilitation (Desai et al. 2021:16; Tambling et al. 2021:1665).

Research shows that the implementation of family involvement in mental health care globally falls far below the recommended levels; hence, there is growing evidence that has been known for decades of beneficial outcomes for both mental health care users (MHCUs) and their families (Eckardt 2022:2). Mental health care users refer to people diagnosed with mental illness who receive care, treatment and rehabilitation of mental health services as an in-patient or outpatient aimed at improving the mental well-being status. In South Africa, long-term mental health care, treatment and rehabilitation are practised and implemented according to the *Mental Health Care Act (MHCA) 17* of the Republic of South Africa (2002:49). The process of rehabilitation includes ‘leave of absence’ (LOA). Section 45 of MHCA alluded that MHCUs can be granted LOA from the mental health institution as pre-discharge planning to prepare family members to stay with their loved ones before discharge from the hospital (South Africa 2002:49). Gowda et al. (2019:707) defined LOA as an observation period, where the family member observes MHCU’s improvement and the responsibilities at home and gives feedback to the mental health care service providers.

Research also shows that mental health care has grown from the asylum system to community care which led to a trend towards involving both MHCUs and their family members in the treatment, care and rehabilitation of mental illness (Ong, Fernandez & Lim 2021:213). The study conducted in Australia by Waller et al. (2019:253) found that engaging the family members encourages both the MHCUs and the health providers to share information and empowers the families to actively participate in the care of their loved ones with mental illness. The study further identified the significance of setting boundaries whereby MHCUs are incapable of making an informed decision as stipulated by the MHCA of the Republic of South Africa (2002) to decide who might be involved in care, treatment and rehabilitation services, and how. In this regard, the study found that the negotiating of clear boundaries should be in place to avoid using family members as the final decision-makers (Waller et al. 2019:253).

What is already known based on the literature is that family involvement is a significant aspect of promoting recovery and well-being of individuals with mental illness (Hansson et al. 2022:16). Furthermore, when the patients’ families are involved as full partners in mental health services, MHCUs, health care providers and families benefit greatly because the quality of mental health care is enhanced. A study conducted in Hong Kong found that social interaction and support from family members are significant aspects that contribute to positive outcomes of mental well-being and recovery for long-term mental health care, treatment and rehabilitation (Lao, Low & Wong 2019:7).

A study conducted in East London, South Africa identified that family involvement was considered a key element of effective intervention to engage families in mental health, but the implementation was a challenge (Mirzaei et al. 2020:813). The same study recommended health care service management needs to consider plans that will actively involve families in mental health to reduce the care burden of family members. Similarly, a conceptual review conducted in Canada, France, the United Kingdom, the United States of America (USA), Germany and Finland identified that family involvement was also considered an effective intervention to involve families in mental health but found it difficult to engage families.

Therefore, the study recommended the basics to clarify why families should be involved in mental health discussed, as well as how mental health providers should perceive family involvement (Dirik et al. 2017:9). According to Shalaby and Agyapong (2020:1), the growing gap that emerged among the MHCUs and health care providers has effectively closed through the implementation of family involvement to provide support in mental health care services. Despite the challenges experienced by health care providers, current literature emphasises the involvement of families in the mental health care workforce (Shalaby & Agyapong 2020).

Research shows that it is important for family members to participate in decision-making on the treatment plan. The studies conducted on family involvement in Western countries revealed that family-centred care is of significance in nursing practice; hence, little is known of nurses’ perceptions when involving family members in mental health care (Alabdulaziz, Moss & Copnell 2017:66). This is in line with a study conducted by Wicks et al. (2018:313) to evaluate the goal of person-centred and family-oriented care and identified the need to scrutinise the impact of patient- and family-centred care that might expand the health inequality gap for MHCUs. However, the study revealed that the presence and participation of family members in mental health promote the sharing of information, partnership and collaboration, as well as negotiations on treatment plans (Wicks et al. 2018:313). Based on the aforementioned literature, the current study sought to describe nurses’ perceptions of involving family members in the care of MHCUs in long-term institutions.

### Research problem statement

There is limited research addressing nurses’ perceptions of family involvement in the care of MHCUs in long-term institutions. Literature supports the importance of family involvement as a key component of effective mental health services. Research on family involvement has been carried out globally for decades in MHIs. In South Africa, there is scanty information on how nurses perceive family participation in mental health care. The author identified that families seldom visit MHCUs and sometimes the family members show frustration, exhaustion and are generally ill at ease with the patients during hospital visits. It was also observed that MHCUs are not acknowledged at home when discharged or granted LOA; hence, they came back to the institution before their expected return dates. Therefore, this article explores and describes nurses’ perceptions of involving family members in the care of MHCUs to identify perceived problems that need improvement in long-term institutions in Limpopo province.

### Conceptual framework

In this study, Thornton’s model of whole-person caring (WPC) was embraced as a framework that is subsequent from theorists in the fields of nursing theories, physical sciences and systems studies (Thornton 2013:4). Additionally, Thornton’s model is further defined as a framework intended to direct health care professionals regarding health and well-being within the health care establishments. Consequently, WPC was originally established to support health professionals in initiating therapeutic environments for both patients and health service providers (Thornton 2013:5). Therefore, it was found that the WPC theory presents realistic influences concerning the critical importance of incorporated body-mind-spirit recovery in a whole-person approach to the scholars (Jonas & Rosenbaum 2021:11). Therefore, in this article, the WPC theory was adopted to address the perceptions of nurses regarding engaging families to actively participate in the care of MHCUs in long-term institutions. Hence, the involvement of the family in mental health could shed light on the fundamental processes of healing and recovery from mental illness through integrating WPC theory (Fulford et al. 2013:63).

## Research methods and design

### Research design

A quantitative descriptive research design was used to examine a phenomenon of interest (Hunter, McCallum & Howes 2019:3). According to Petrongolo and Toothaker (2021:2), a descriptive quantitative study was designed to explore the perceptions about the nurses that draw the researcher’s attention. In this regard, the author quantifies the perceptions of nurses regarding engaging family members to actively participate in the care of MHCUs in long-term MHIs in Limpopo province.

### Setting

This study was conducted at selected rural MHIs in Vhembe, Mopani and Capricorn Districts of the Limpopo province in South Africa. Each institution admits less than 400 MHCUs (Mopani 235, Vhembe 354 and Capricorn 365). All three community MHIs, in Limpopo, in the mentioned districts provide long-term mental health care for MHCUs diagnosed with substance-induced psychotic disorders, schizophrenia, depressive disorders, anxiety disorders, intellectual disability and physical disability from rural areas. In addition, mental health services are also offered in primary health care settings including mobile clinics which were not included in the study.

### Population, sampling and sample size

The total population comprised 1005 nurses working in the long-term MHIs in Limpopo province. Therefore, the sample encompasses 91 (7%) from 217 nurses working at Evuxakeni, 103 (8%) from 251 in Hayani and 166 (19%) from 537 in Thabamoopo MHIs. Probability simple random sampling was used to select nurses from Vhembe, Mopani and Capricorn Districts of Limpopo province to generate a sample, hence generalising the population. The sample size was calculated from the three MHIs, 360 (34%) from 1005 nurses of which were drawn from three hospitals. All categories of nursing staff who were directly providing mental health services to the MHCUs and were on duty during data collection, both males and females of all ages were included. This article presents the quantitative section of the mixed method study to the qualitative articles published from the PhD thesis (Mabunda, Mangena-Netshikweta, Lebese & Olaniyi 2022; Mabunda, Netshikweta & Lebese 2023). In addition, this article is unique as it focuses on the quantitative descriptive design from the same thesis mentioned in the acknowledgement.

### Data collection

Data were collected using questionnaires with closed-ended items and were designed in English, to accommodate all nurses based on the review of the literature. To identify errors, a pilot study of 15 nurses working at Vembe District was conducted and not included in the project. Closed-ended, self-administered questionnaires were used to gather data from nurses on duty. The questionnaire had two sections. Section A required the demographic information of participants. Hammer (2011:261) adds that the inclusion of demographic information greatly adds to the understanding of universal propositions and variants that exist among populations. Therefore, demographic information was required from respondents. Section B contained self-administered questions about nurses’ perceptions of involving family members in the care of MHCUs in long-term MHIs. Three hundred and eighty questionnaires were handed out to nurses on duty. A total of 368 questionnaires were received of which 360 were completed and eight were incomplete. Of the total of 380 questionnaires handed out to the nurses, 12 were missing. Data were collected from June to August 2017. In addition, a qualified professional nurse with a Master’s degree in Nursing was assigned as a research assistant. The service of a research assistant was sought to assist with the distribution and collection of questionnaires. Furthermore, the training of research assistant was done 2 weeks before data collection.

### Data analysis

The Statistical Package for the Social Science (SPSS) version 23 was used to analyse the data. The questionnaire comprises 17 self-assessment questions for nurses’ perceptions of engaging family members to actively participate in mental health was developed and attached as a supplementary file. The study results were presented in frequency and percentages. The results were presented in the form of tables to illustrate the frequency and percentages. The discussion of results was supported by references from relevant literature sources, where applicable. Data obtained from the pilot were not included in the main data collection.

### Validity and reliability

Face validity was observed to avoid bias and to confirm the instrument measured the characteristics that it was designed to measure. The author reviewed the literature to acquire information about nurses’ perceptions of involving family members in mental health and obtained critique from mental health experts when developing the questionnaire. The author consulted the biostatistician in developing the questionnaire to confirm that the instrument measures what it is supposed to measure. The questionnaire was reviewed by a biostatistician before data collection to enhance the quality of the study (Singh 2017:800; Taherdoost 2016:2). A Likert scale matrix with four response options (strongly agree, agree, disagree and strongly disagree) was used to measure responses from the respondents in frequency and percentage as indicated in Table 1. In addition, a dichotomous scale was used to collapse data into a two-point scale to block respondents’ opportunity to be neutral to a question (Kusmaryono, Wijayanti & Maharani 2022:626). The author ensured that at face value, the questionnaire appeared to be a relevant measure of the content under discussion in the study (Sileyew 2019:3; Story & Tait 2019:198; Surucu & Maslakçi 2020:2696). Validity was ensured by presenting the research instruments to six experts in the field of mental health care services to evaluate the contents within the instruments. Reliability was ensured by conducting a pilot study of 15 nurses working at Vembe District not included in the project, to identify errors.

**TABLE 1:** Demographic data of participants.

Respondents	Characteristics	*n* (*N* = 360)	%
Years of service (years)	< 2	21	5.8
	2–5	58	16.1
	6–10	105	29.1
	11–30	103	28.6
	> 30	73	10.3
Rank	Enrolled nursing auxiliary	150	41.7
	Enrolled nurse	53	14.7
	Professional nurse	142	39.4
	Operational managers	13	3.6
	Nursing service managers	2	6.0
Qualification	Certificate	187	51.9
	Diploma	104	28.9
	Degree	67	18.6
Age (years)	20–25	10	2.8
	25–35	98	27.2
	35–45	149	41.4
	45–55	74	20.6
	> 55	29	8.1
Gender	Male	76	21.1
	Female	284	78.8
Marital status	Single	165	45.8
	Married	170	47.2
	Divorced	11	3.1
	Widowed	14	3.9

### Ethical considerations

Permission to conduct the current study was obtained from the University of Venda’s Higher Degrees Committee, project numbers: SHS/16/PDC/35/1611), and reference number (LP/4/2/2) was received from the provincial Department of Health of the Limpopo province. Permission was also obtained from authorities of the Mopani, Vhembe and Capricorn Districts, as well as from the managers of Evuxakeni, Hayani and Thabamoopo MHIs. The author observed the right to privacy, beneficence, non-maleficence, autonomy and self-determination through voluntary participation through informed consent. The researcher avoided asking questions that could affect respondents psychologically regarding their fears, personal views as well as actions that cause harm intentionally. The respondents were informed not to put their names on any questionnaire. They were also reassured that all information would be treated confidentially. Finally, the researcher ensured that the research assistant signed a confidentiality agreement with the first author to distribute and collect questionnaires before data collection.

## Results

Demographic data of participants are summarised in Table 1 and will be presented first.

Table 1 shows the demographic data description of respondents to allow the readers to determine to whom the research findings are generalised and the comparisons to be made across replications of studies. The majority of the respondents worked between 6 and 10 years with 29.1% (*n* = 105), 11 and 30 years with 28.6% (*n* = 103) and only 21 respondents (5.8%) working less than 2 years. The responses showed the highest number of 51.9% (*n* = 187) respondents with certificates that include enrolled nurses and this shows that 28.9% (*n* = 104) diplomas and 16.1% (*n* = 69) degrees were attained by professional nurses.

However, the age characteristics of respondents, 41.4% (*n* = 149), were between 35 years to 45 years showing the majority of nurses were adults with experience in nursing. The current study showed the highest number 78.8% (*n* = 284) of female respondents and 21.1% (*n* = 76) of males. In addition, the marital status characteristic was found to have the highest number of 47.2% (*n* = 170) of respondents who were married, 45.8% (*n* = 165) of respondents were single, 3.1% (*n* = 11) were divorced and 3.9% (*n* = 14) were widowed.

### The nurses’ perceptions regarding family involvement in caring for mental health care users

This section aims to describe nurses’ perceptions regarding family involvement in caring for MHCUs in long-term MHIs in Limpopo province. The results were interpreted separately. However, some responses with similar items were grouped together to facilitate the interpretation of the findings. The researcher identified related items as the key findings to present the results logically based on the information from responses as indicated in Table 2. These include challenges regarding family involvement, referrals of MHCUs to the relevant health care provider as well as family visits during MHCUs’ admission in the MHIs.

**TABLE 2:** Responses regarding family involvement.

Responses	Agree (*N* = 360)		Disagree (*N* = 360)	
	*n*	%	*n*	%
**Challenges regarding family involvement**				
There are challenges regarding involvement of the family members in mental health care	313	86.9	47	13.1
Insufficient family involvement	329	91.4	31	8.6
Poor family contact affects the provision of mental health care services	290	80.6	70	19.4
MHCUs’ family contact numbers available	57	15.8	303	84.2
MHCUs when granted LOA came back to the hospital on the expected date	351	97.5	9	2.5
MHCUs accepted at home when granted LOA or discharged	332	92.2	28	7.8
MHCU absconds from home to the hospital when granted LOA	26	7.2	334	92.8
**Referrals of MHCUs to the relevant health care provider**				
Cases referred to the relevant health care provider	317	88.1	43	11.9
Feedback provided indicates family members’ contact available, ready to be involved	185	51.3	175	48.6
MHCUs who did not have identity documents or birth certificates during admission to MHIs were referred to social workers	258	71.7	102	28.3
MHCU who died without family contact, cases of death were referred to social workers for family tracing	334	92.8	26	7.2
MHCUs buried as paupers without tracing family members	16	4.5	344	95
**Family visits during MHCUs’ admission in the MHIs**				
MHCUs in your unit have families who gradually visit	126	35	234	65
There is good interaction between the MHCUs and their family members	111	30.8	249	69.1
MHCUs being happy when visited by their family members	355	98.6	5	1.4
Family members’ attitude positive when health care providers conduct home visit	110	30.6	250	69.7
Family members are involved in cases of medical illnesses and procedures	314	87.2	46	12.8

MHCU, mental health care users; MHI, mental health institutions; LOA, leave of absence.

### The responses regarding family involvement

Table 2 shows responses regarding family involvement. Similar items were grouped together to make the presentation of results easier.

### Challenges regarding family involvement

Out of 360 respondents, 86.9% (*n* = 313) confirmed that there were challenges regarding the involvement of family members in mental health care, while 13.1% (*n* = 47) disagreed. Although 91.4% (*n* = 329) of the respondents indicated that challenges included insufficient family involvement, 8.6% (*n* = 31) disagreed. However, 80.6% (*n* = 290) of the respondents agreed that poor family contacts affected the provision of mental health care services while 19.4% (*n* = 70) disagreed. Out of the 360 respondents, 15.8% (*n* = 57) confirmed that all of the MHCUs in their facility had family contacts, whereas 84.2% (*n* = 303) indicated that some of the MHCUs had family contacts.

Out of the 360 respondents, 97.5% (*n* = 351) proved that some of the MHCUs reported back on the expected date after an LOA. Only 2.5% (*n* = 9) indicated that MHCUs back to the hospital before the expected date when granted LOA. The study indicates the highest number 92.2% of respondents (*n* = 332) who reported that some of the MHCUs went for LOA when granted, and 7.8% (*n* = 28) reported that families refused to accept LOA for MHCUs. Twenty-six respondents (7.2%) said that some of the MHCUs absconded from home to the hospital when granted LOA, and 92.8% (*n* = 334) indicated that no MHCUs absconded from home back to the hospital when granted LOA.

### Referrals of mental health care to the relevant health care provider

A total of 88.1% (*n* = 317) indicated that cases of poor family contacts were referred to the relevant health care providers while 11.9% (*n* = 43) disagreed. About 83.6% (*n* = 301) of the respondents confirmed that family members do visit MHCUs in the hospital. Fifty-one per cent agreed (*n* = 185) that health care provider feedback was provided indicating that family contacts were available and family members were willing to become involved and 48.63% (*n* = 175) disagreed. A total of 71.7% of respondents (*n* = 258) confirmed that MHCUs who did not have identity documents or birth certificates during admission to MHIs were referred to social workers. However, 22.8% (*n* = 82) indicated that family members were notified to provide MHCUs’ identity documents or birth certificates. The majority 92.8% of the respondents (*n* = 334) indicated that cases of death were referred to social workers for family tracing while 7.2% (*n* = 26) disagreed. Additionally, 4.5% of respondents (*n* = 16) reported that MHCUs were buried as paupers without tracing family members while 95% (*n* = 344) disagreed.

### Family visits during mental health care users’ admission in the mental health institutions

Out of 360 respondents, 35% (*n* = 126) indicated that MHCUs have families who gradually visit and 65% (*n* = 234) disagreed. Despite 30.8% of respondents (*n* = 111) confirming that good interactions occur between the MHCUs and their families, 69.1% (*n* = 249) indicated that there was a poor interaction between MHCUs and their families. The majority 98.6% of respondents (*n* = 355) indicated that MHCUs were happy when visited by family members while 1.4% (*n* = 5) disagreed. The current study found that a high number of MHCUs were happy when visited by family members. In terms of the family members’ attitudes when the hospital health care providers conduct home visits were positive, 69.7% (250) respondents indicated that family members have positive attitudes when health care providers conduct home visits and 30.6% (110) disagreed. In addition, the majority 87.2% of respondents (*n* = 314) confirmed that in cases of medical illnesses and procedures, family members were involved. However, 12.8% (*n* = 46) disagreed.

## Discussion

The current article described nurses’ perceptions of involving family members in the care of MHCUs to promote active participation of families with mentally ill patients in long-term MHIs in Limpopo province. Despite the WPC theory emphasising nurses’ role in empowering MHCUs recovery, WPC theory is embraced to address the nurses’ perceptions of involving families in the care of their loved ones with mental illness while admitted to long-term institutions. It is therefore noted that integrating WPC theory to engage families of individuals diagnosed with mental illness to actively participate in mental health could shed light on the important measures to promote recovery from mental illness (Fulford et al. 2013:63; Leger et al. 2023:8).

The demographic distribution by profession in the current study includes years of service, rank, qualifications, age and gender of nurses who participated in the study as indicated in Table 1. Demographic data were relevant for this study to understand whether research respondents are representative of the general population (Chow et al. 2022:2).

The current study found that the majority of respondents worked for 6–10 and 11–30 years in the MHIs. The number of years indicates that most of the nurses have work experience which may helped them to improve nurses’ competencies and perceptions of inequality of mental health care services (Jia et al. 2021:43). South African Nursing Council (SANC) (2018:2) gender statistics bring out 257 542 female and 28 162 male nurses in Limpopo province. Gender was also important in this study, as it helped to identify variances from both sexes that could undertake the responsibility of coordinating and monitoring families to actively participate in mental health as males and females have different views (Huang et al. 2020:125). The reason for the higher number of female nurses could be that nursing has often been regarded as a female profession (Van der Cingel & Brouwer 2021:4).

Qualification was also important in this study, as it helped to identify variances from different competency levels and also contributes to the quality of mental health care services (Rizany, Hariyati & Handayani 2018:155). Additionally, age and marital status characteristics were found to have the highest numbers of respondents also reflecting the maturity and extent of their perceptions of involving family members in long-term MHIs. Therefore, demographic data was an important part of this study and was used to examine the quantifiable statistics of a particular population since the trends in demographics historically change in a population over time (Connelly 2013:267).

The responses regarding family involvement showed that there are challenges in mental health care. Most of the respondents reported that the challenges include insufficient family involvement and poor family contact that hinders the provision of MHC services. This finding is similar to Huang et al. (2020:121) study that revealed the significance of engaging families of individuals diagnosed with mental illness is essential. Hence, there are challenges in facilitating sufficient interaction between patients and their families concerning treatment plans. It was noticed from the previous studies that nurses have a crucial role in facilitating interaction between patients and their families and require competence and resources to reduce the challenge for quality mental health services (Haavisto et al. 2021:583).

A high percentage of cases of poor family contact are referred to by the relevant health care provider as well as by the families. This concurs with a Namibian study of Makki et al. (2018:2921) that showed that involving family members in MHC helped families to understand the nature of social support and the significance of family relationships. However, family involvement seemed to be pivotal to any care, treatment and rehabilitation efforts; hence, intervention includes the use of information from families that promotes the well-being of the distressed and ensures that supportive environments are fostered (Makki et al. 2018:2921; Schueller et al. 2019:248). A total of 301 (83.6%) nurses admitted that families are not visiting MHCUs in the hospital. Similarly, the current study found that families’ involvement is poor, perhaps because the current study was conducted in rural areas.

The responses regarding family contacts revealed that there are MHCUs who do not have family contacts. The absence of family contacts revealed that there are MHCUs who cannot be visited at all; hence, the possibility of involving the families is very low in such situations. The literature revealed that family contacts are crucial in the provision of health care services. Authors recommended that families should be accessible physically and telephonically to accept home visits by hospital health care providers before the patients’ discharge from the hospital to help families be prepared for the caregiving role (Cadel et al. 2022:7; Gilliss, Pan & Davis 2019:24). The current study found instances where family members’ contacts were available, but family members have reasons for not ready to be involved. The reasons could be distance and transport money to travel to the hospital might be a challenge. Such cases were referred to social workers for intervention.

In addition, the current study showed that family members are not ready to be involved for various reasons. Studies used to investigate patient- and family-related risk factors for posttraumatic stress in family members of chronic critical illness patients, revealed that the availability of family members’ phone contacts helps health professionals in case there is a need to make arrangements to meet the families for treatment plan purposes (Hart et al. 2020:95; Roen et al. 2018:3).

The current study further showed that there are MHCUs who have not been visited since admission because the social workers were failing to find their families. However, in the current study, few respondents added that there are MHCUs who were being buried as paupers. Burns et al. (2014:912) reported that health professionals in Canada experienced challenges in developing and implementing the mechanisms for supporting MHCUs to interact with their families. The processes and activities that provide opportunities for families to be involved in meaningful interactions might enhance meaningful discussions and decision-making concerning their loved ones with mental illness when admitted in the MHIs. (Doherty et al. 2019:4).

It is possible that the MHCUs are being buried as paupers because their families were not found when they were traced. However, Moriarty, Steils and Manthorpe (2019:10) confirmed that, in instances where MHCUs died without family contact, the case is also referred to the relevant health care providers for family tracing before burial. However, caring for MHCUs who die without family contact being present psychologically affects nurses when they think of a patient being a pauper (Lake et al. 2022:803). According to Totman et al. (2015:505), when a patient dies in the hospital without family contact, it is the responsibility of the social workers to ensure that family members are traced to bury their loved ones and to express their grieving feelings and emotions. Therefore, facilitation is needed towards MHCUs being buried as paupers (Totman et al. 2015:505; Witt et al. 2020:2).

In addition, poor interaction between MHCUs and families was confirmed by most respondents and shows that family involvement is poor in such situations. Therefore, the current study found that family members do not want to be involved in mental health care. Most respondents confirmed that the attitude of family members during home visits was an indication that they do not accept their loved ones having mental illnesses. Few respondents indicated that family members’ attitudes during home visits were positive. Furthermore, Subu et al. (2021:3) found that family members display a negative attitude when visited by mental health professionals. However, the respondents indicated that the attitude of family members seems to be neutral. The evidence that MHCUs are not accepted by their families was shown by most respondents who confirmed that MHCUs come back before the expected return date when granted LOA (Bakali, Du Plessis & Froneman 2023:7). According to Watson and Choo (2021:8), LOA is a significant pre-discharge therapeutic intervention that is proposed to increase patient excellence of life in the community. It was found that LOA reduces long-term hospitalisation; hence, it supports MHCUs to live meaningful lives in the community during discharge. In addition, it was emphasised that LOA is an approach towards patient-centredness to shared decision-making on MHCUs’ ability to cope at home (Barlow & Dickens 2018:648).

The current study further indicated that there are MHCUs who did not even go home when granted LOA or who were discharged because of family’s attitude towards mental illness. A similar finding was made by Subu et al. (2021:1), who suggested that hands-on experience could be a powerful way to change families’ attitudes towards MHCUs and to promote involvement through formal family therapy. As a result, initiatives to improve patient and public involvement should address the knowledge, attitudes and skills of staff at all levels of the service (Subu et al. 2021:1). However, the current study shows that families who do not accept mental illness at all contribute to MHCUs returning to the hospital before the expected date when granted LOA.

The current study found that some of the MHCUs abscond from home back to the hospital when granted LOA. A study to explore why patients prefer to stay in the hospital revealed that patients with low socioeconomic status prefer hospitalisation than those with high socioeconomic status. The study further highlighted that patients unnecessarily return to the hospital because they feel that being at home is chaotic; hence, the situation at home prompted several MHCUs to return to the hospital for readmission (Balaban et al. 2015).

Little is known of the MHCUs absconding from home back to the hospital when granted LOA. However, MHCUs abscond from home after realising that the hospital system can provide all their basic needs (Moorkath, Vranda & Naveenkumar 2018:476). The current study further showed a high percentage of MHCUs being happy when visited by family members, which reveals that MHCUs appreciate the presence of their families in such situations. Therefore, the fact that MHCU reported being happy when visited indicates that MHCUs appreciate support from their families and social interactions while sharing their thoughts and feelings (Jovanović, Campbell & Priebe 2019:13; Richards et al. 2019:40). In addition, when patients’ family members visit their loved ones in the hospital, they will be most involved in the patient’s treatment and care decisions (Macdonald 2021:30).

In the current study, the respondents confirmed that some MHCUs do not have identity documents or birth certificates. In this regard, patients’ health records, including information about their identity documents, are significant during patients’ examinations, such as treatment, actions and other services provided to patients in different health care facilities. Hence, this minimises de-identifying psychiatric intake records (Davison et al. 2021:6). The current study indicated a high percentage of respondents who agreed that, in cases of medical illness and procedures, family members are involved. However, some of the respondents disagreed that families are involved in cases of medical illness and procedures. A study on analysing barriers faced by physicians when providing health care revealed that physicians have a role to ensure that the families’ capability for medical decision-making or informed consent, and that medical consent is reached. In addition, the consent of legal guardian(s) should be facilitated effectively to inform families about medical procedures (Thannhauser, Morris & Gamble 2021:377).

This study found that this can happen to MHCUs who do not have family contacts, as the social worker also failed to find family members. However, caring for MHCUs who die without family contact psychologically affects nurses when they think of the patient being a pauper (Lake et al. 2022:803). Therefore, nurses have the responsibility to collaborate with the social worker to facilitate towards MHCUs being buried as paupers. (Totman et al. 2015:505; Witt et al. 2020:2).

## Conclusion

The nurses’ perception of family involvement in long-term MHIs in Limpopo province revealed that there are challenges that hinder the quality provision of mental health care services. These challenges include insufficient family involvement, poor family contact that affects the provision of mental health care services, cases to the relevant health care provider and failure by family members to visit MHCUs in the hospital. However, the provision of quality mental health care, treatment and rehabilitation implies that families should be involved in interacting with MHCUs and discussing how mental illness affects their lives. Finally, the current study identified areas that hinder the effectiveness of quality mental health care services such as families not being willing to be involved in mental health care, families not visiting their loved ones during hospitalisation, MHCUs not being accepted by families when granted LOA or discharge, MHCUs returned to the hospital before expected date from LOA, as well as MHCUs absconding from home to the hospital when granted LOA. Further studies should be conducted to include families’ perspectives of MHCUs in long-term MHIs to determine the factors to promote family involvement and support.

### Strengths and limitations

The response rate for this study was low, with 6% of all nurses working from three MHIs who responded. The author conducted spot checks comparing information recorded on checklists to check whether all aspects of the questionnaire were fully completed. In addition, of the 380 questionnaires handed to the nurses, eight questionnaires were incomplete, to the extent that they did not provide appropriate results; hence, 12 were missing. Therefore, 360 questionnaires confined the outcome of the study. In addition, the study was limited to nurses working in general hospitals psychiatric wards or units admitting MHCUs in the same districts.
